# Fe_2_O_3_/Ni Nanocomposite Electrocatalyst on Cellulose for Hydrogen Evolution Reaction and Oxygen Evolution Reaction

**DOI:** 10.3390/ijms242216282

**Published:** 2023-11-14

**Authors:** Sadhasivam Thangarasu, Nimisha Baby, Mrunal Bhosale, Jaeman Lee, Changseong Jeong, Tae-Hwan Oh

**Affiliations:** Department of Chemical Engineering, Yeungnam University, Gyeongsan 38541, Republic of Koreamrunal.snst.1@gmail.com (M.B.); ljm9390@yu.ac.kr (J.L.); csjeong555@naver.com (C.J.)

**Keywords:** water splitting, hydrogel, electrocatalysts, binder, oxygen evolution reaction, hydrogen evolution reaction, thickening agent, catalyst, hydrogen energy

## Abstract

A key challenge in the development of sustainable water-splitting (WS) systems is the formulation of electrodes by efficient combinations of electrocatalyst and binder materials. Cellulose, a biopolymer, can be considered an excellent dispersing agent and binder that can replace high-cost synthetic polymers to construct low-cost electrodes. Herein, a novel electrocatalyst was fabricated by combining Fe_2_O_3_ and Ni on microcrystalline cellulose (MCC) without the use of any additional binder. Structural characterization techniques confirmed the formation of the Fe_2_O_3_–Ni nanocomposite. Microstructural studies confirmed the homogeneity of the ~50 nm-sized Fe_2_O_3_–Ni on MCC. The WS performance, which involves the hydrogen evolution reaction (HER) and the oxygen evolution reaction (OER), was evaluated using a 1 M KOH electrolyte solution. The Fe_2_O_3_–Ni nanocomposite on MCC displayed an efficient performance toward lowering the overpotential in both the HER (163 mV @ 10 mA cm^−2^) and OER (360 mV @ 10 mA cm^−2^). These results demonstrate that MCC facilitated the cohesive binding of electrocatalyst materials and attachment to the substrate surface. In the future, modified cellulose-based structures (such as functionalized gels and those dissolved in various media) can be used as efficient binder materials and alternative options for preparing electrodes for WS applications.

## 1. Introduction

“Hydrogen energy” is a clean synthetic fuel that provides energy without harmful emissions (hydrogen fuel cell—Anode: 2H_2_ → 4H^+^ + 4e^−^; Cathode: 4H^+^ + 4e^−^ + O_2_ → 2H_2_O; Overall: 2H_2_ + O_2_ → 2H_2_O + Energy). Electrochemical hydrogen generation via water splitting (WS) has received considerable interest among the numerous available hydrogen production techniques [1,2,3]. In WS, two half-reactions occur: the hydrogen evolution reaction (HER) at the cathode and the oxygen evolution reaction (OER) at the anode. The characteristics and functionality of the electrocatalyst play a major role in the electrochemical WS process. Electrocatalysts based on platinum-group metals (PGMs) exhibit excellent OER (IrO_2_, RuO_2_) and HER (Pt) performances owing to their abundant active sites and intrinsic properties that facilitate interactions with various species [4,5]. However, their high manufacturing costs and scarcity are major drawbacks [5,6]. Therefore, more cost-effective electrocatalysts for WS have been developed as alternatives. Non-precious-metal-based materials, such as Ni, Fe, and Co-based compounds, are recognized as excellent electrocatalysts owing to their abundant active sites and high electrical conductivity [7,8,9]. Fe_2_O_3_ is widely regarded as a promising candidate for electrocatalytic applications because of its cost-effectiveness, abundance, and remarkable chemical stability [10]. Ahmad et al. developed a heterostructure electrocatalyst comprising Fe_2_O_3_ and FeP, capable of attaining an overpotential of 264 mV at 10 mA cm^−2^ for the OER [11]. Multi-metal oxides, such as Fe_2_O_3_-ZnCo_2_O_4_ featuring Fe_2_O_3_ as a MOF, display efficient overall WS [12]. Under alkaline conditions, Fe_2_O_3_ combined with MnO can catalyze the OER with an overpotential of 370 mV at 10 mA cm^−2^ [13]. Notably, the use of Fe_2_O_3_-based nanocomposite structures, including Ni and its compounds, has been shown to significantly boost OER and HER performances [14,15]. Compounds based on Ni have been shown to significantly increase the reaction kinetics of the OER [7]. Fe_2_O_3_ and Ni-based compounds show remarkable synergistic effects in reducing reaction barriers and augmenting reaction rates because of their exceptional interfacial properties [7,8,16]. The incorporation of Ni nanoparticles into various electrocatalysts has shown significant improvements in overall water-splitting efficiency. This is attributed to the inherent catalytic activity of Ni, which enhances the reaction kinetics and thus enhances the performance of water-splitting processes [17,18,19,20].

Recently, innovative approaches that utilize various bio-derived materials for WS systems have been established [21,22,23]. Bio-derived materials are regarded as viable candidates for electrodes and membranes in various energy conversion and storage systems [24,25,26,27]. The most important advantages of bio-derived materials are their abundance, low cost, and ease of use. Different types of bio-derived materials have been used to prepare electrocatalysts for various energy systems. To facilitate the WS process, bio-based materials undergo carbonization to enhance their electrical conductivity and specific surface area characterized by porosity [28,29]. To further enhance the electrocatalytic behavior of bio-derived electrocatalysts, various metal derivatives have been incorporated and deposited on their surfaces and pores in recent years. Interestingly, the incorporation of heteroatoms into modified bio-based materials can also enhance their electrochemical performance. Alternatively, bio-derived materials can be used as binders in electrode materials for various energy and environmental device systems [24,30,31,32]. Bio-derived materials possessing thickening and binding characteristics are more suitable as binder materials for electrode preparation. In addition, most bio-derived materials have abundant functional groups on their surfaces that facilitate species transport, which enhances electrochemical reaction kinetics [33]. Among the different types of bio-derived materials, cellulose-based derivatives have attracted considerable attention owing to their favorable intrinsic properties, such as the presence of abundant hydroxyl groups, which enhance intermolecular and intramolecular hydrogen bonding, and their amenability to chemical modification that alter their properties [34]. 

Binder materials for electrode manufacturing must be electrochemically inactive, be highly mechanically stable, and undergo minimal volume change [35]. Synthetic polymers, such as perfluorosulfonic acid, polyvinylidene difluoride and polytetrafluoroethylene, are typically used as binders in the development of various types of electrodes as they provide stability over extended periods of operation. However, synthetic polymers has certain disadvantages, such as toxicity and insolubility in water, which necessitate the use of organic solvents, leading to detrimental environmental impact [35]. Binder materials that are soluble in water or exhibit water-holding behavior, particularly adsorption, and possess functional groups that potentially enhance electrochemical reactions by facilitating ion transport and reducing the distance between the electrochemical species and electrode surfaces are desirable. In addition, the incorporation of the hydrophilicity nature of materials into the electrode has the potential to efficiently stimulate electrochemical processes [36]. From this perspective, cellulose-based materials, such as microcrystalline cellulose (MCC) and its derivatives, can be considered as substrates, dispersing agents, or binder materials for electrocatalyst and electrode preparation [31,33,35,37]. The uniform integration of electrically conductive elements on the cellulose surface is a viable approach for the synthesis of hydrogel-based electrocatalysts after appropriate modifications. Alternatively, increasing the amount of electrocatalyst materials on the cellulose surface can improve electrode efficacy. If cellulose has the required features, particularly in terms of thickening and binding behavior, it has the potential to effectively link various electrocatalytic materials. Consequently, it can be used directly in the fabrication of electrode materials. Herein, a novel electrocatalyst material was developed by combining Fe_2_O_3_ and Ni with MCC. The structural and microstructural properties of the source and as-prepared materials were characterized by Fourier-transform infrared spectroscopy (FT-IR), X-ray diffraction (XRD), X-ray photoelectron spectroscopy (XPS), scanning electron microscopy (SEM), transmission electron microscopy (TEM), and energy-dispersive X-ray spectroscopy (EDS). The homogenous dispersion and loading of the nanocomposite materials on MCC were confirmed through microstructural studies. WS (HER and OER) performances were evaluated using 1 M KOH electrolyte. The Fe_2_O_3_ and Ni nanocomposite on MCC provided lower overpotential both in the HER (163 mV@ 10 mA cm^−2^) and OER (360 mV @ 10 mA cm^−2^).

## 2. Results and Discussion

**Figure 1:**
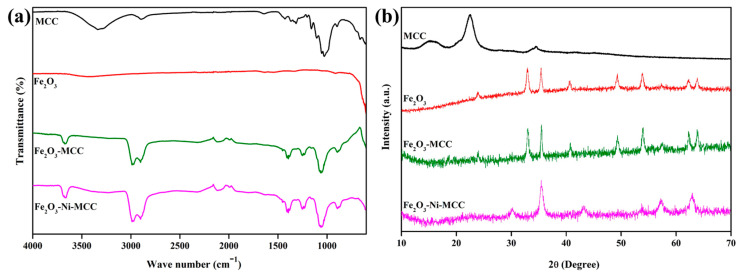
(**a**) FT-IR and (**b**) XRD of the MCC, Fe_2_O_3_, Fe_2_O_3_-MCC, and Fe_2_O_3_-Ni-MCC.

Cellulose materials are considered efficient candidates (multi-component materials) for various energy systems owing to their favorable functional properties, such as the presence of many hydroxyl functional groups, abundance, wettability, low production cost, and ease of structural modification [25,26,38,39,40]. The effects of MCC as a binding agent on the efficacy of WS electrodes were investigated. FT-IR and XRD analyses were performed to investigate the functional group properties and crystallinity, respectively, of the as-received MCC. The FT-IR spectrum of MCC is shown in Figure 1a. The prominent peak at 3332 cm^−1^ is indicative of the presence of the hydroxyl (–OH) functional groups in the cellulose material. The most prominent peaks appeared at 1027 and 1052 cm^−1^, corresponding to the stretching vibration of the C–O bond. Additionally, the peaks positioned at 2895, 1640, 1428, 1159, and 896 cm^−1^ were attributed to C–H, O–H, CH_2_ stretching vibration of C–O-C and C–H, respectively [41,42,43]. The FT-IR spectrum confirmed the existence of a cellulose structure. Furthermore, the as-developed electrocatalysts were analyzed using FT-IR spectroscopy before and after the incorporation of MCC. Figure 1a shows the FT-IR spectra of the Fe_2_O_3_ and Fe_2_O_3_–Ni samples, both with and without MCC. Fe_2_O_3_–MCC gave rise to a greater number of peaks than pristine Fe_2_O_3_. A wide prominent peak ranging from 3200 to 3500 cm^−1^ consistently appeared for all samples, indicating the presence of –OH functional groups. In Fe_2_O_3_–MCC, the additional peaks positioned at 2901, 1640, 1056, and 892 cm^−1^ correspond to C–H stretching, O–H vibration, C–O stretching, and CH vibration, respectively [42,44]. An analogous pattern was observed in the Fe_2_O_3_–Ni composite after the incorporation of MCC. The FT-IR spectra verified the existence of MCC in conjunction with the electrocatalyst materials. Furthermore, a shift in the peak of the composite material containing MCC was observed, which could be attributed to the intermolecular interaction between the functional groups on the MCC surface and the electrocatalyst materials. XRD analysis was performed to conduct a more comprehensive assessment of the crystalline characteristics of the MCC and electrocatalyst material, and the results are shown in Figure 1b. Figure 1b shows the XRD spectra of MCC, which are in agreement with the reported MCC pattern (JCPDS#03289) [45,46]. The strongest peak appeared at 2θ = 22.50°. Additional peaks were observed at 2θ = 15.01°, 16.31°, and 34.52°, corresponding to the lattice planes of (1–10), (110), and (004), respectively [47,48]. The XRD pattern confirms that MCC exhibits crystalline behavior. For Fe_2_O_3_, the peaks at 2θ values of 23.94°, 32.98°, 35.43°, 40.58°, 49.27°, 53.87°, and 62.30° correspond to the lattice planes of (012), (104), (110), (113), (024), (116), (214), and (300), respectively. Successful α-Fe_2_O_3_ synthesis was confirmed based on the comparison of the acquired XRD pattern with JCPDS Card No.00-033-0664 [49,50,51]. For Fe_2_O_3_-MCC, a similar characteristic peak of α-Fe_2_O_3_ was attained. Furthermore, it is worth noting that the peaks corresponding to Fe_2_O_3_ were present subsequent to the decoration of Fe_2_O_3_ on the surface of MCC. The results confirmed the effective production of a composite consisting of Fe_2_O_3_ and MCC. In Fe_2_O_3_-Ni-MCC, the peaks at 2θ values observed at 30.14°, 35.43°, 43.17°, 53.75°, 57.24°, and 63.04° correspond to the lattice planes of (220), (311), (400), (422), (511), and (440), respectively. The growth of Fe_2_O_3_ in conjunction with Ni for Fe_2_O_3_-Ni-MCC demonstrated the presence of the crystal phase of γ-Fe_2_O_3_, which was assigned the JCPDS card number 00039-1346 [51,52]. Furthermore, two additional peaks were observed for Ni together with γ-Fe_2_O_3_ in Fe_2_O_3_–Ni–MCC. Moreover, the primary MCC peak was observed in the Fe_2_O_3_–Ni–MCC composite material at 2θ = 22.4°. The additional peaks observed in the spectra were smaller owing to the presence of a smaller amount of MCC in the Fe_2_O_3_–Ni–MCC composite. These results verified the homogeneity of the Fe_2_O_3_–Ni electrocatalyst materials in conjunction with MCC. Based on the analyses of the structural characteristics, it can be confirmed that the electrocatalyst was successfully synthesized and integrated with the MCC structure, resulting in a promising composite electrode material. 

**Figure 2:**
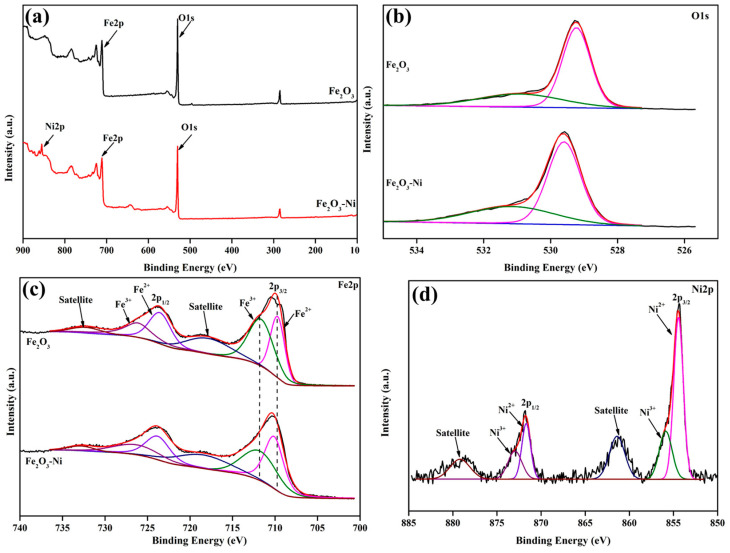
XPS analysis of the Fe_2_O_3_ and Fe_2_O_3_-Ni electrocatalysts. (**a**) Survey spectra and deconvoluted spectra of (**b**) O1s, (**c**) Fe2p, and (**d**) Ni2p for the electrocatalysts.

The as-prepared electrocatalyst materials were analyzed using XPS to determine their elemental compositions and chemical and electronic states. The XPS survey spectra of the Fe_2_O_3_ and Fe_2_O_3_–Ni electrocatalysts are shown in Figure 2a. For Fe_2_O_3_, the most dominant peaks were at ~530.1 and ~711 eV, which were related to O 1s and Fe 2p, respectively. The presence of these distinctive peaks indicated the formation of Fe_2_O_3_. Furthermore, an additional peak corresponding to Ni 2p (~855.1 eV) was observed for Fe_2_O_3_–Ni, along with the O 1s and Fe 2p peaks. This observation confirmed the successful incorporation of Ni with Fe_2_O_3_, thereby validating the synthesis of the Fe_2_O_3_–Ni electrocatalyst. The deconvoluted oxygen XPS profiles are shown in Figure 2b. The peaks observed at approximately 529.4 eV indicated the presence of lattice oxygen (Fe-O) [53,54]. The Fe-deconvoluted XPS spectra (Figure 2c) contained two prominent peaks corresponding to Fe 2p_3/2_ and Fe 2p_1/2_. Additionally, two satellite peaks were observed at 718.7 and 732.6 eV [55,56]. These results confirmed the production of Fe_2_O_3_ in both cases. The additional Ni peaks in the Fe_2_O_3_–Ni sample were deconvoluted and are shown in Figure 2d. Two primary and additional satellite peaks were identified. The observed peaks located at 855.4 and 871.2 eV can be attributed to the presence of Ni^2+^ (Ni 2p_3/2_ and Ni 2p_1/2_, respectively) [57,58]. The satellite peaks located at 861.4 and 876.2 eV correspond to Ni 2p_3/2,sat_ and Ni 2p_1/2,sat_, respectively. The XPS results verified the successful fabrication of Fe_2_O_3_ and Fe_2_O_3_–Ni electrocatalysts and their composition. 

**Figure 3:**
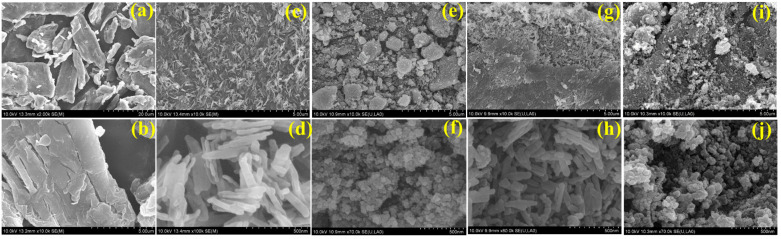
Scanning electron microscope images of (**a**,**b**) MCC, (**c**,**d**) Fe_2_O_3_, (**e**,**f**) Fe_2_O_3_-Ni, (**g**,**h**) Fe_2_O_3_-MCC, and (**i**,**j**) Fe_2_O_3_-Ni-MCC.

SEM was used to examine the surface characteristics of the MCC and as-prepared electrocatalysts with and without MCC to determine their morphology, size, and shape. Figure 3 shows the SEM images of MCC, Fe_2_O_3_, Fe_2_O_3_–Ni, Fe_2_O_3_–MCC, and Fe_2_O_3_–Ni–MCC. As shown in Figure 3a,b, the dimensions of the as-received MCC were within the range of a few micrometers and were largely uniform. Smaller MCC crystals were detected alongside larger ones. In addition, MCC exhibited a structure similar to that of interconnected MCC. The as-prepared Fe_2_O_3_ electrocatalysts present a cylindrical morphology, as shown in Figure 3c,d. The mean length of Fe_2_O_3_ was estimated to be approximately 250 nm, whereas the mean diameter was approximately 50 nm. Furthermore, the shape and size of the Fe_2_O_3_ electrocatalysts were consistent. The uniformity and comparable dimensions of the nanomaterials are beneficial for the development of electrocatalysts intended for WS applications. Figure 3e,f demonstrate the formation of a distinct morphological configuration of Fe_2_O_3_–Ni in comparison to that of Fe_2_O_3_ without Ni. The coexistence of Fe_2_O_3_ and Ni led to change the Fe_2_O_3_ crystal phase and significant alterations in the surface morphology, particularly in terms of shape and size. The Fe_2_O_3_–Ni nanoparticles exhibited a spherical morphology. In addition, the as-developed Fe_2_O_3_–Ni electrocatalyst materials are in the form of an accumulation. In contrast to Fe_2_O_3_, rod-like structures were not observed in Fe_2_O_3_–Ni. The successful integration of Ni with Fe_2_O_3_ resulted in the production of diverse surface morphologies, such as nanoparticles. The size of Fe_2_O_3_ bearing Ni was approximately 50 nm, as shown in Figure 3f. Furthermore, the particles were of a consistent size across the structure. Notably, the particles exhibited a high degree of interconnectedness. The SEM images of the Fe_2_O_3_ and Fe_2_O_3_–Ni electrocatalysts with MCC are shown in Figure 3g–j, respectively. As shown in Figure 3g, the Fe_2_O_3_–MCC composite exhibited a cylindrical rod-like morphology, where Fe_2_O_3_ was present on the MCC structure. The Fe_2_O_3_ nanoparticles were effectively immobilized and uniformly distributed over the entire surface of the MCC. The extensive interactions between MCC and Fe_2_O_3_ are beneficial for the development of electrode materials. Furthermore, the morphology and dimensions of Fe_2_O_3_ remained unchanged when combined with MCC, as shown in Figure 3h. Similar behavior was observed for the interaction between Fe_2_O_3_–Ni and MCC in the case of Fe_2_O_3_–Ni–MCC. As shown in Figure 3i, the Fe_2_O_3_–Ni composite was effectively adhered to MCC. It is noteworthy that the Fe_2_O_3_–Ni electrocatalyst material effectively coated the entire surface of MCC, exhibiting a remarkable level of interaction between the two materials. Furthermore, the dimensions of the electrocatalysts remained mostly unaltered throughout their integration with MCC because they were anchored onto the MCC surface. The SEM results showed that the dimensions of the electrocatalyst materials were within the nanometer range. Furthermore, electrocatalyst materials were efficiently integrated and attached to the MCC surface. In Fe_2_O_3_-Ni-MCC, the smaller Fe_2_O_3_ with Ni nanomaterial is able to effectively increase OER and HER performances because it has a higher specific and active surface area than the larger Fe_2_O_3_ nanomaterial in Fe_2_O_3_-MCC. 

**Figure 4:**
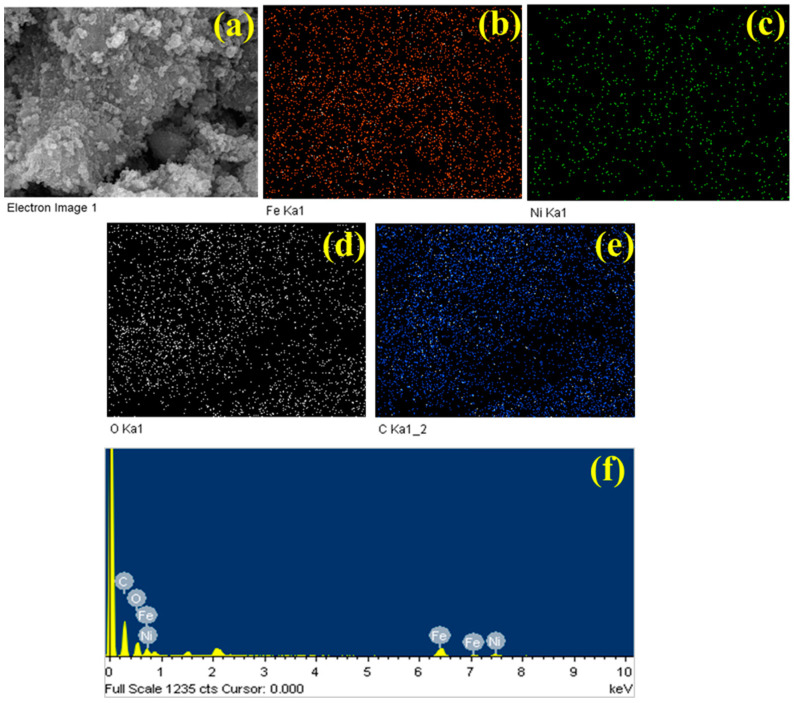
EDS mapping analysis of Fe_2_O_3_-Ni-MCC. (**a**) Micrograph image, elements of (**b**) Fe, (**c**) Ni, (**d**) O, (**e**) C, and (**f**) EDS spectra results.

To gain insight into the distribution of the components in the multi-component composite and the uniformity of the electrocatalyst materials inside the composite, EDS mapping was conducted. The EDS mapping data and corresponding spectra of Fe_2_O_3_, Fe_2_O_3_–Ni, Fe_2_O_3_–MCC, and Fe_2_O_3_–Ni–MCC are presented in Figures S1–S3 and Figure 4, respectively. The elemental composition of Fe_2_O_3_ indicated the presence of Fe and O, confirming its successful synthesis. The coexistence of Ni, Fe, and O was observed for Fe_2_O_3_–Ni, as shown in Figure S2. The presence of Ni in the composite electrocatalysts verified its homogenous integration with Fe_2_O_3_. Figure S3 provides visual evidence of the successful decoration and interaction of the Fe_2_O_3_ electrocatalyst with MCC. It is evident that MCC was effectively covered by the Fe_2_O_3_ material. Figure 4 depicts the mapping study conducted on the Fe_2_O_3_–Ni–MCC composite. As shown in Figure 4a, the electrocatalyst materials were efficiently immobilized and interconnected on the surface of the MCC substrate. Figure 4b–e show the distribution of Fe, Ni, O, and C, respectively, in Fe_2_O_3_–Ni–MCC. Based on the EDS mapping data, it can be concluded that the synthesis of a homogenous nanocomposite electrocatalyst was successful and that the electrocatalysts exhibited strong interconnectivity with the MCC structure. To substantiate the existence of elements inside the nanocomposites, the obtained EDS spectra confirmed their presence in all of the electrocatalyst materials. Fe, Ni, O, and C were detected in Fe_2_O_3_–Ni–MCC, further supporting the formation of Fe_2_O_3_–Ni–MCC, as shown in Figure 4f. The EDS mapping and spectral findings indicated the formation of Fe_2_O_3_, Fe_2_O_3_–Ni, Fe_2_O_3_–MCC, and Fe_2_O_3_–Ni–MCC, which can be effectively utilized directly as electrode materials for WS applications.

**Figure 5:**
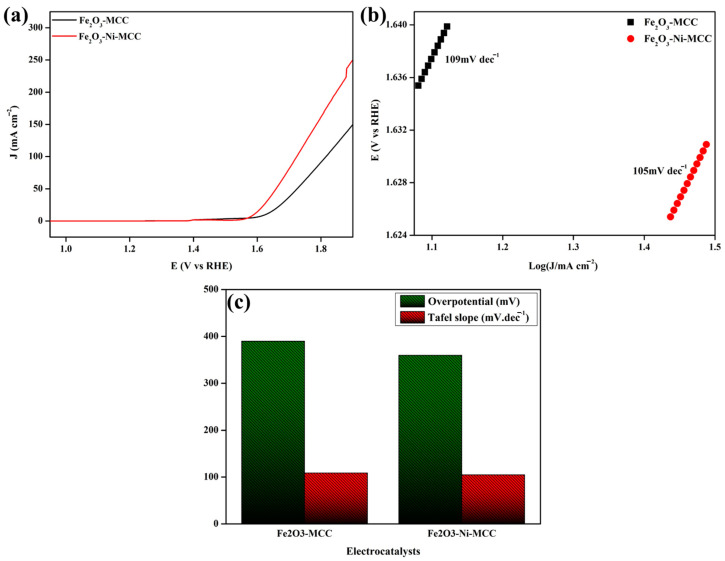
Electrochemical characterizations of OER performances: (**a**) LSV curves at 5 mV/s scan rate, (**b**) corresponding Tafel slopes, (**c**) comparison of Fe_2_O_3_-MCC and Fe_2_O_3_-Ni-MCC HER performance concerning overpotential at 10 mA cm^−2^ and Tafel slope.

To assess the electrochemical efficacy of the electrocatalyst materials on MCC, their OER performance was evaluated under alkaline conditions in 1 M KOH solution. The catalytic activities of Fe_2_O_3_–MCC and Fe_2_O_3_–Ni–MCC were assessed by depositing them on the surface of the electrocatalyst support without using other supplementary binder materials. Linear sweep voltammetry (LSV) was used to evaluate the effectiveness of the electrode materials for the OER. In the LSV study, the working electrode consisted of the active materials coated on Ni foam together with MCC. The counter electrode was a Pt plate, and the reference electrode was Hg/HgO. The LSV measurements were conducted at a scan rate of 5 mV s^−1^. The OER polarization curves of Fe_2_O_3_–MCC and Fe_2_O_3_–Ni–MCC are shown in Figure 5a. Electrocatalysts incorporating MCC demonstrated notable electrocatalytic activity. These results clearly demonstrate that the use of MCC is beneficial for the cohesive binding of electrocatalyst materials and their successful attachment to the substrate surface. Thus, MCC plays a crucial role in anchoring the materials on the surface and promoting their interaction during electrochemical processes. Furthermore, the electrocatalyst materials remained adhered to the substrate surface during the OER. Fe_2_O_3_–MCC exhibited notable OER performance, as evidenced by an overpotential of 396 mV at a current density of 10 mA cm^−2^. The incorporation of Ni into Fe_2_O_3_–MCC improved the electrochemical performance. As shown in Figure 5a, the Fe_2_O_3_–Ni–MCC catalyst achieved a current density of 10 mA cm^−2^ for the OER with an overpotential (η_10_) of 360 mV. Here, the interaction between Ni and Fe_2_O_3_ resulted in a synergistic effect, which is the most advantageous factor for reducing the overpotential in the OER. To understand the OER kinetics of the electrocatalyst materials, Tafel plots, derived from the OER polarization curve, were constructed. Figure 5b shows the Tafel plots of Fe_2_O_3_–MCC and Fe_2_O_3_–Ni–MCC. The Tafel slope of Fe_2_O_3_–Ni–MCC (105 mV dec^−1^) was lower than that of Fe_2_O_3_–MCC (109 mV dec^−1^). The lower Tafel slope and overpotential of Fe_2_O_3_–Ni–MCC (Figure 5c) implies that it exhibits better reaction kinetics than Fe_2_O_3_–MCC because it requires a lower activation energy for reaction completion. Ni-based materials exhibit a dynamic electronic structure that may significantly enhance intermediate reactions during the OER, specifically, the conversion of OH* to OOH*. The OER performance of Fe_2_O_3_–Ni–MCC is comparable to those reported in other studies [11,12,13,14,51,59,60,61,62,63,64].

**Figure 6:**
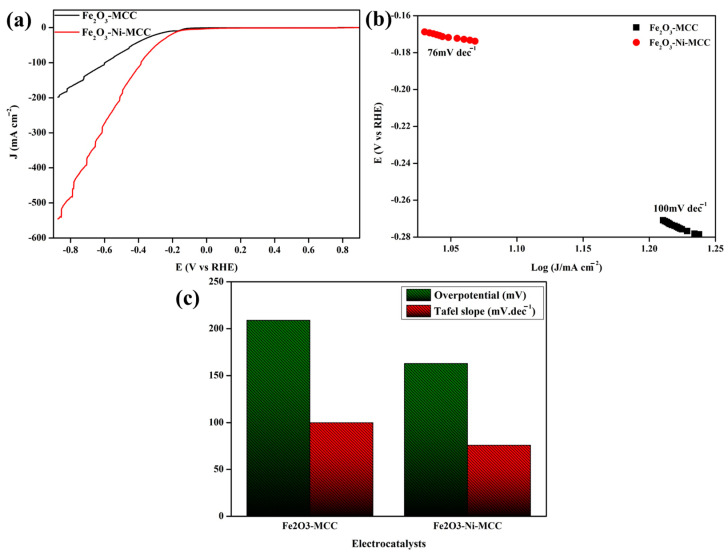
Electrochemical characterizations of HER performances: (**a**) LSV curves at 5 mV/s scan rate, (**b**) corresponding Tafel slopes, (**c**) comparison of Fe_2_O_3_-MCC and Fe_2_O_3_-Ni-MCC OER performance concerning overpotential at 10 mA cm^−2^ and Tafel slope.

To evaluate the electrocatalyst performance for the HER, LSV analysis was carried out in 1 M KOH. The LSV performances of Fe_2_O_3_–MCC and Fe_2_O_3_–Ni–MCC were evaluated under similar conditions at a scan rate of 5 mV/s (Figure 6a). The combination of the Fe_2_O_3_ electrocatalyst and MCC demonstrated significant electrochemical HER activity. The HER data obtained for the Fe_2_O_3_–MCC system indicate an overpotential of 209.5 mV at a current density of 10 mA cm^−2^. Compared with Fe_2_O_3_–MCC, Fe_2_O_3_–Ni–MCC showed superior electrocatalytic HER activity. A current density of 10 mA cm^−2^ was achieved by the Fe_2_O_3_–Ni–MCC system with a potential of 163 mV. The coexistence of two distinct materials in the electrocatalyst results in a synergistic effect, thereby enhancing the HER performance. For a more comprehensive analysis of the effects of the electrocatalyst materials, Tafel plots were generated based on the polarization curves obtained from the HER experiments. The Tafel slope was determined to validate the reaction kinetics of the electrocatalysts. Figure 6b,c show the Tafel plots and the results of the Fe_2_O_3_–MCC and Fe_2_O_3_–Ni–MCC reactions, respectively. The Tafel slope of Fe_2_O_3_–Ni–MCC (76 mV dec^−1^) was lower than that of Fe_2_O_3_–MCC (100 mV dec^−1^). Therefore, Fe_2_O_3_–Ni–MCC exhibited improved reaction kinetics compared to Fe_2_O_3_–MCC, indicating that it requires a lower activation energy for the HER. The HER performance of Fe_2_O_3_–Ni–MCC is comparable to that of previously reported electrocatalysts [12,61,62,63,65]. 

**Figure 7:**
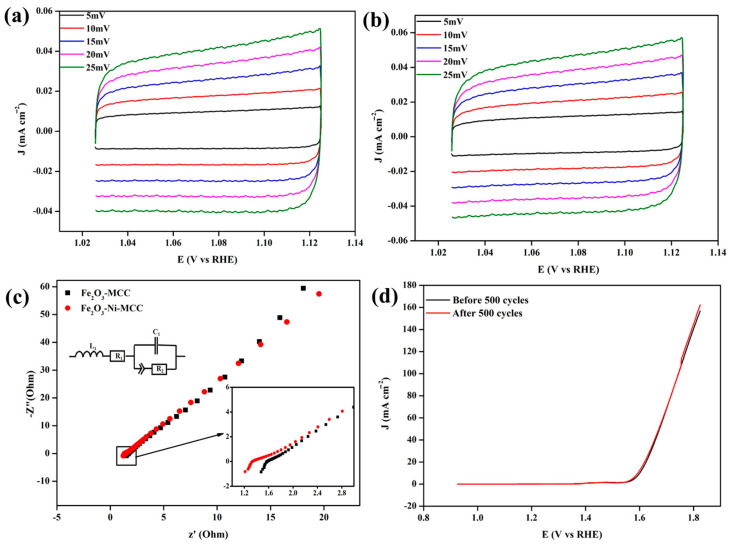
CV analysis of (**a**) Fe_2_O_3_-MCC and (**b**) Fe_2_O_3_-Ni-MCC at different scan rates; (**c**) EIS analysis results of Fe_2_O_3_-MCC and Fe_2_O_3_-Ni-MCC; (**d**) LSV; (OER) performances of Fe_2_O_3_-Ni-MCC before and after 500 CV cycles.

To gain a better understanding of the influence of the electrocatalyst material, cyclic voltammetry (CV) measurements were conducted in 1 M KOH. To determine the active surface area, CV experiments were conducted within a non-faradaic region at scanning rates of 5, 10, 15, 20, and 25 mV/s. The CV of Fe_2_O_3_–MCC and Fe_2_O_3_–Ni–MCC are shown in Figure 7a,b, respectively. Fe_2_O_3_–Ni–MCC exhibited a greater active surface area than Fe_2_O_3_–MCC. Fe_2_O_3_–Ni–MCC achieved efficient performance, primarily owing to effective adsorption and desorption of active intermediates and facilitated by the presence of a greater number of active sites in its structure. Furthermore, owing to its inherent functional characteristics, MCC facilitated the adsorption and desorption of active intermediates. Therefore, MCC has the capacity to effectively evaluate the preparation of electrodes for WS systems. The Fe_2_O_3_–MCC and Fe_2_O_3_–Ni–MCC samples were subjected to electrochemical impedance spectroscopy (EIS) analysis to determine their solution resistance and charge transfer efficiency. The electrochemical impedance spectra of both materials were obtained within a frequency range of 100 kHz–0.1 Hz at a fixed amplitude of 10 mV. Figure 7c displays the Nyquist plots and equivalent circuit models for the Fe_2_O_3_–MCC and Fe_2_O_3_–Ni–MCC systems. Compared to the Fe_2_O_3_–MCC system, Fe_2_O_3_–Ni–MCC exhibited lower charge transfer resistance. The efficient HER and OER activity of Fe_2_O_3_–Ni–MCC can largely be attributed to the accelerated reaction rate between the electrocatalyst and electrolyte during the electrochemical process. The stability of Fe_2_O_3_–Ni–MCC was assessed by conducting LSV measurements at a scan rate of 5 mV/s for the OER, both before and after 500 CV cycles. The comparison of the Fe_2_O_3_–Ni–MCC LSV curves before and after 500 CV cycles is shown in Figure 7d. Based on the obtained data, it can be concluded that the Fe_2_O_3_–Ni–MCC system exhibits superior durability, making it suitable for long-term applications. Moreover, the beneficial effects of using MCC as a binder were confirmed. MCC effectively anchored the materials on the surface and facilitated and maintained their interconnectivity throughout the electrochemical performance. During the OER, the electrocatalyst materials did not detach from the surface of the substrate, owing to the robust MCC binding. Furthermore, MCC facilitated the adsorption and desorption of active intermediates during electrochemical processes, owing to its inherent functional properties. As a result, it has been shown that the use of MCC as a binder has the potential to significantly influence the electrochemical performances of both HER and OER. Numerous investigations need to be conducted with a variety of novel electrode materials for a variety of electrochemical systems, such as water splitting, fuel cells, batteries, CO_2_ reduction, and NO_x_ reduction systems [66,67,68,69], in order to further explore the impact and possibilities of cellulose and other bio-derived materials ass binders to prepare the electrodes, from a commercial perspective. These investigations will allow for further exploration of the impact and possibilities of cellulose and other bio-derived materials as binders to prepare the electrodes.

## 3. Conclusions

In this study, a new type of electrocatalyst was prepared by combining a Fe_2_O_3_–Ni nanocomposite with MCC. The developed materials were coated onto the electrode surface via a facile process without using an additional binder. FT-IR, XRD, and XPS characterization techniques were used to determine the functional and structural properties of the as-received MCC and as-prepared Fe_2_O_3_, Fe_2_O_3_–Ni, Fe_2_O_3_–MCC, and Fe_2_O_3_–Ni–MCC materials. SEM and EDS mapping were used to determine the homogeneity of the as-developed Fe_2_O_3_–Ni–MCC materials. It was confirmed that the as-prepared Fe_2_O_3_–Ni materials were firmly attached to MCC. The HER and OER performances of Fe_2_O_3_–MCC and Fe_2_O_3_–Ni–MCC were evaluated using 1 M KOH electrolyte solution. The HER and OER activities of the Fe_2_O_3_–Ni nanocomposite on MCC were the highest. At 10 mA cm^−2^, the observed overpotential for the OER and HER were 360 and 163 mV, respectively. The effective binding of electrocatalyst materials and their excellent connection to the MCC surface were clearly demonstrated. These results suggest that MCC plays a significant role in promoting adhesion between materials and maintaining their cohesion throughout electrochemical operations. 

## 4. Materials and Methods

Nickel (II) nitrate hexahydrate (Ni(NO_3_)_2_⋅6H_2_O, 97%), sodium hydroxide (NaOH), dimethyl sulfoxide (C_2_H_6_SO, 99%), N-methyl 2-pyrrolidone (NMP, 99%), and hydrochloric acid (HCl, Extra pure) were obtained from Duksan Chemicals, Republic of Korea. Iron (III) chloride hexahydrate (FeCl_3_⋅6H_2_O, 98%), potassium hydroxide (KOH, >85%), and ethyl alcohol (EtOH, 94.5%) were obtained from Daejung Chemicals and Metals, the Republic of Korea. Acetylene black (˃99.9%) was purchased from Alfa Aesar. Microcrystalline cellulose (MCC, 20 μm) was obtained from Sigma Aldrich. Nickel foam was purchased from NARA Cell-Tech Corporation. All the chemicals used in this study were used without any additional purification. Deionized (DI) water was used throughout the experiment.

The electrocatalyst materials were prepared as follows: FeCl_3_⋅6H_2_O was dissolved in 50 mL of deionized (DI) water to a final concentration of 0.1 M and stirred for 30 min. Then, 16 mL of 2.0 M NaOH was added dropwise to the above-mentioned solution with constant stirring until the pH reached 12. Subsequently, Ni(NO_3_)_2_⋅6H_2_O (0.05 M) was added to the final solution. Then, the reaction mixture was transferred to a Teflon-lined stainless steel autoclave, and the reaction was performed at 180 °C for 15 h. After the reaction was complete, the autoclave was naturally cooled to room temperature, and the as-prepared product was thoroughly washed multiple times with DI water. Finally, the as-prepared material was dried in an oven at 80 °C overnight and then annealed at 500 °C for 1 h. The as-prepared sample was denoted as Fe_2_O_3_–Ni nanocomposite. To prepare the Fe_2_O_3_ nanostructures [11], a similar procedure was followed without the Ni source material. To prepare Fe_2_O_3_–Ni–MCC, the prepared Fe_2_O_3_–Ni (40 mg) was added to 5 mg of dissolved MCC and stirred for 2 h. Before mixing Fe_2_O_3_–Ni and MCC, both materials were effectively dispersed. The final product was then dried at 50–80 °C for 12 h.

The presence of functional groups in the as-received and as-prepared materials was confirmed by FT-IR analyses (Perkin Elmer FT–IR spectrometer (Spectrum 100, Shelton, CT, USA)). The formation and crystallinity of the materials were confirmed using XRD (Xpert Pro, Malvern, UK). The chemical states of the as-developed materials were investigated using XPS (Thermo scientific K-α surface analysis). The surface morphology, distribution, and elemental composition of the electrocatalyst materials were determined using SEM with EDS (Hitachi S-4800, Ibaraki, Japan). The particle sizes of the as-prepared materials were measured using TEM (Hitachi H-7600, Ibaraki, Japan). 

Ni foam (1 cm × 1 cm) pretreated with 3 M HCl, DI water, and EtOH and dried overnight at 60 °C was used for preparing the electrode. The desired ratio of Fe_2_O_3_–Ni–MCC (45 mg) to acetylene black (5 mg) was mixed homogeneously, and a slurry was prepared to coat the Ni foam surface. The active-material-coated Ni foam was flattened using a compressor (30 s). Finally, the working electrode was dried overnight at 60 °C. To evaluate the electrocatalyst performance, a three-electrode system comprising working (active material-coated Ni foam), reference (Hg/HgO), and counter (platinum plate) electrodes was used. N_2_-saturated 1 M KOH was used as the electrolyte solution for electrochemical measurements. LSV was used to assess the HER and OER performances of the electrocatalysts in the potential ranges of 0.0 to −1.8 V and 0.0 to 1.0 V, respectively, at a scan rate of 5mV/s. CV experiments were performed in 1 M KOH to further understand the active surface area of the electrocatalysts at varying scan rates. EIS was performed from 100 kHz to 0.1 Hz with a fixed amplitude of 10 mV.

## Data Availability

Data are contained within the article.

## Supplementary Materials

The following supporting information can be downloaded at https://www.mdpi.com/article/10.3390/ijms242216282/s1.
