# Supplementary material for: Fe2O3/Ni Nanocomposite Electrocatalyst on Cellulose for Hydrogen Evolution Reaction and Oxygen Evolution Reaction
Source: Int J Mol Sci. 2023 Nov 14;24(22):16282. doi: 10.3390/ijms242216282 (PMC10671088; doi:10.3390/ijms242216282)
Supplement: Supplementary file 1 [file ijms-24-16282-s001.zip › ijms-2681625-supplementary.pdf]

International Journal of *Molecular Sciences*

Supporting Information

MDPI

Article

Construction of Fe_2O_3/Ni nanocomposite electrocatalyst on the cellulose as a binder for hydrogen evolution reaction and oxygen evolution reaction

Sadhasivam Thangarasu *,†, Nimisha Baby †, Mrunal Bhosale, Jae Man Lee, Chang Seong Jeong and Tae Hwan Oh*

Department of Chemical Engineering, Yeungnam University, Gyeongsan 8541, Republic of Korea; sadhasivam.nano@yu.ac.kr (S.T.); mishababy099@gmail.com (N.B.); mrunal.snst.1@gmail.com (M.B.); ljm9390@yu.ac.kr (J.M.L.); csjeong555@naver.com (C.S.J.); taehwanoh@ynu.ac.kr (T.H.O.)

* Correspondence: sadhasivam.nano@gmail.com and sadhasivam.nano@yu.ac.kr (S.T.); taehwanoh@ynu.ac.kr (T.H.O.)

† These authors contributed equally to this work.

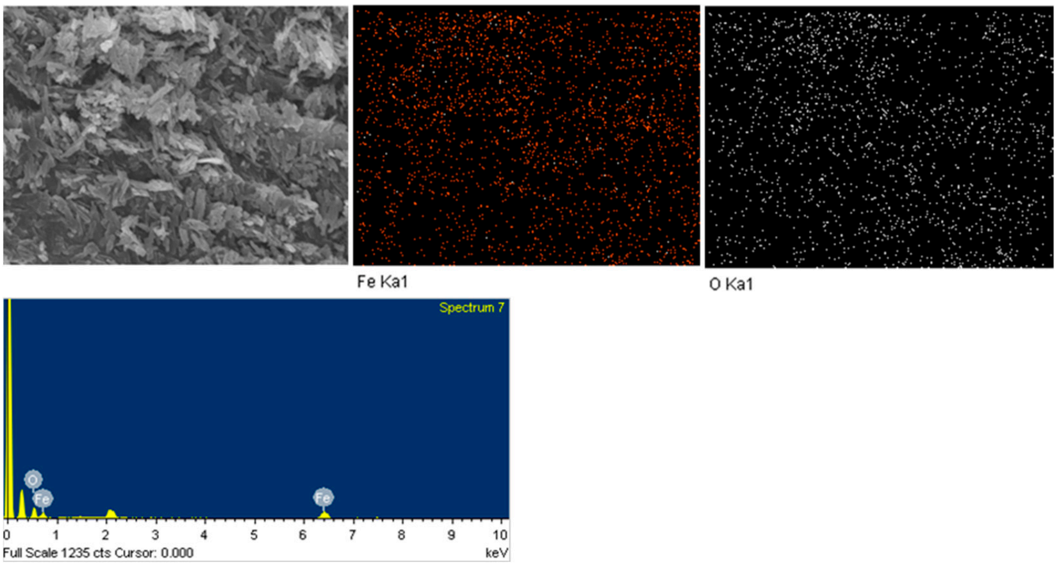

Figure S1: EDS mapping and spectra of Fe_2O_3.

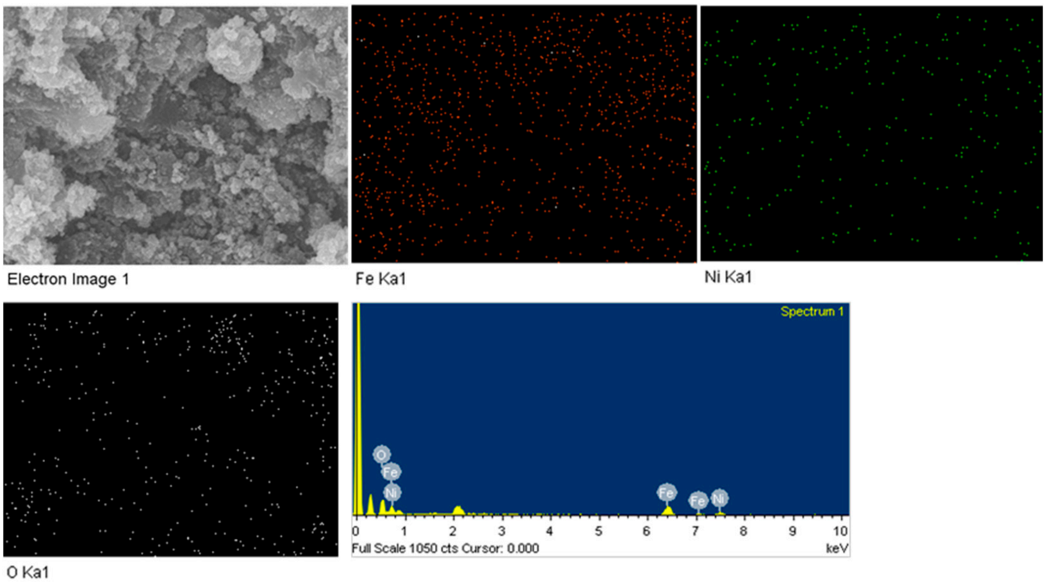

Figure S2: EDS mapping and spectra of Fe_2O_3-Ni.

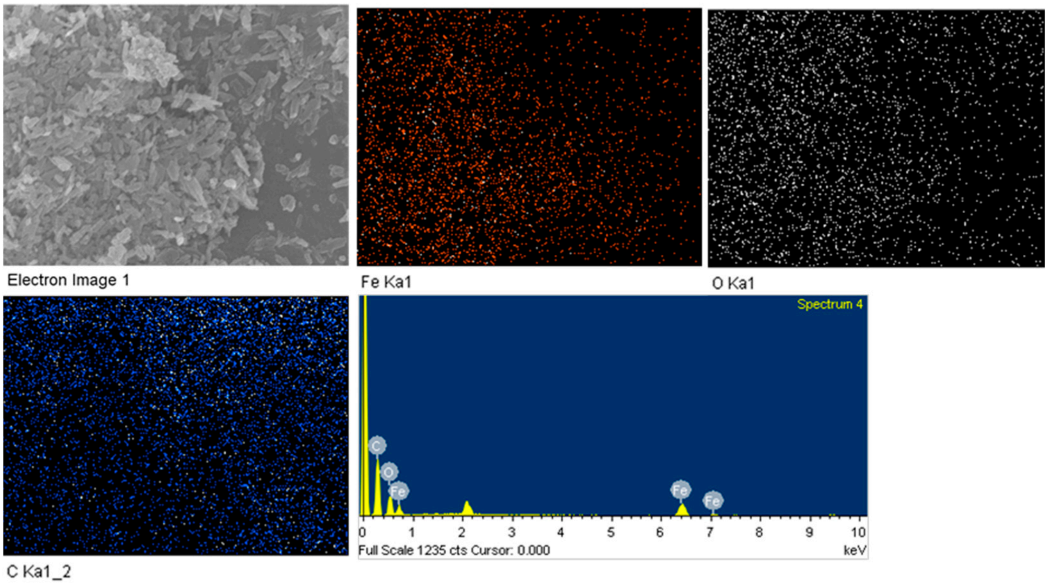

Figure S3: EDS mapping and spectra of Fe_2O_3-MCC.
